# Chemical Composition and Antioxidant Activity of Essential Oils from *Eugenia patrisii* Vahl, *E. punicifolia* (Kunth) DC., and *Myrcia tomentosa* (Aubl.) DC., Leaf of Family Myrtaceae

**DOI:** 10.3390/molecules26113292

**Published:** 2021-05-29

**Authors:** Celeste de Jesus Pereira Franco, Oberdan Oliveira Ferreira, Ângelo Antônio Barbosa de Moraes, Everton Luiz Pompeu Varela, Lidiane Diniz do Nascimento, Sandro Percário, Mozaniel Santana de Oliveira, Eloisa Helena de Aguiar Andrade

**Affiliations:** 1 Faculdade de Química, Universidade Federal do Pará, Rua Augusto Corrêa S/N, Guamá, Belém 66075-900, PA, Brazil; celeste.frango12@gmail.com (C.d.J.P.F.); angeloquimica17@gmail.com (Â.A.B.d.M.); eloisa@museu-goeldi.br (E.H.d.A.A.); 2 Programa de Pós-Graduação em Biodiversidade e Biotecnologia- Rede Bionorte, Universidade Federal do Pará, Rua Augusto Corrêa S/N, Guamá, Belém 66075-900, PA, Brazil; oberdan@museu-goeldi.br (O.O.F.); evertonlpvalerla@gmail.com (E.L.P.V.); percario@ufpa.br (S.P.); 3 Programa de Pós-Graduação em Química, Universidade Federal do Pará, Rua Augusto Corrêa S/N, Guamá, Belém 66075-900, PA, Brazil; 4 Laboratório Adolpho Ducke–Coordenação de Botânica, Museu Paraense Emílio Goeldi, Av. Perimetral, 1901, Terra Firme, Belém 66077-830, PA, Brazil; lidianenascimento@museu-goeldi.br; 5 Laboratório de Pesquisas em Estresse Oxidativo, Universidade Federal do Pará, Rua Augusto Corrêa S/N, Guamá, Belém 66075-900, PA, Brazil

**Keywords:** myrtaceae, natural products, essential oils, antioxidant capacity

## Abstract

Essential oils (EOs) were extracted from *Eugenia patrisii*, *E. punicifolia*, and *Myrcia tomentosa,* specimens A and B, using hydrodistillation. Gas chromatography coupled with mass spectrometry (GC/MS) was used to identify the volatile constituents present, and the antioxidant capacity of EOs was determined using diphenylpicryl-hydrazyl (DPPH) and trolox equivalent antioxidant capacity (TEAC) assays. For *E. patrisii*, germacrene D (20.03%), bicyclogermacrene (11.82%), and (*E*)-caryophyllene (11.04%) were identified as the major constituents of the EOs extracted from specimen A, whereas specimen B primarily comprised γ-elemene (25.89%), germacrene B (8.11%), and (*E*)-caryophyllene (10.76%). The EOs of *E. punicifolia* specimen A contained β-Elemene (25.12%), (*E*)-caryophyllene (13.11%), and bicyclogermacrene (9.88%), while specimen B was composed of (*E*)-caryophyllene (11.47%), bicyclogermacrene (5.86%), β-pinene (5.86%), and γ-muurolene (5.55%). The specimen A of *M*. *tomentosa* was characterized by γ-elemene (12.52%), germacrene D (11.45%), and (*E*)-caryophyllene (10.22%), while specimen B contained spathulenol (40.70%), α-zingiberene (9.58%), and γ-elemene (6.89%). Additionally, the chemical composition of the EOs was qualitatively and quantitatively affected by the collection period. Furthermore, the EOs of the studied specimens, especially specimen A of *E. punicifolia*, showed a greater antioxidant activity in DPPH rather than TEAC, as represented by a significantly high inhibition percentage (408.0%).

## 1. Introduction

Aromatic and medicinal plants have been used in food, agriculture, and the treatment of diseases for many years [1]. They are known for producing essential oils (EOs) and impart fragrances or aromas that stimulate the sense of smell. Usually a product of secondary metabolism, EOs are of great economic importance and have applications in several fields such as pharmaceuticals, cosmetics, and food. They are present in different parts of the plant including flowers, leaves, stems, fruits, branches, and seeds [2,3,4].

EOs are complex, hydrophobic mixtures primarily composed of monoterpenes, sesquiterpenes, and their oxygenated derivatives [5,6]. They are high-value products with a wide variety of interesting biological properties. These include antifungal, antibacterial, anticancer, cytotoxic, and allelopathic properties with profound effects on animals, humans, and even other plants [7].

The Myrtaceae family of angiosperms includes approximately 130 genera and 5671 species, distributed in tropical and subtropical regions of the planet, with centers of diversity in South America, Australia, and tropical Asia [8]. In Brazil, the Myrtaceae family comprises 27 genera and 1026 species and is distributed across five regions and different phytogeographic domains [9]. Scattered in the Brazilian forests, the species of this family are economically important and cultivated not only for their edible fruits but also for ornamental and purposes and as a source of timber [10]. In addition, they are sources of EOs that have insecticidal, parasiticidal, antifungal, antibacterial, antimicrobial, and antioxidant properties [11,12].

Eugenia is one of the most important genera of the Myrtaceae family, with edible fruits, wood, and EOs being commercially exploited in addition to its use in traditional medicine [13,14]. In Brazil, this genus is represented by 392 species distributed across all regions [15]. *Eugenia patrisii,* popularly known as Ubaía-rubí, predominantly grows in the Amazon [15] and produces edible fruits that are used to make juice, jam, and ice cream [16]. *Eugenia punicifolia* (Kunth), DC. is a member of the Pedra-ume-caá genus and is used in traditional medicine to treat diabetes, fever, and other ailments in the form of infusions [17,18].

*Myrcia* is also one of the largest genera within the Myrtaceae family, with over 400 species found in different biomes from the south to the north of Brazil [19]. Members of the genus *Myrcia* exhibit several biological activities, including antinociceptive, anti-inflammatory, antioxidant, antimicrobial, hypoglycemic, and anti-hemorrhagic activities. Many *Myrcia* species also produce EOs with a high concentration of mono- and sesquiterpenes, as well as extracts rich in phenolic compounds and flavonoids, responsible for a wide range of biological activities [10].

The primary aim of this study was to determine the chemical composition of EOs extracted from *Eugenia patrisii*, *E*. *punicifolia*, and *Myrcia tomentosa* specimens, and evaluate their antioxidant activity, to contribute to the studies on aromatic plants found in the Amazon region, particularly in the state of Pará, Brazil.

## 2. Results and Discussion

### 2.1. Yields of Essential Oils

The EOs content of *E*. *patrisii*, was 0.24% for specimen A and 0.77% for specimen B when calculated on a dry basis. Specimens A and B of *E*. *punicifolia* had 0.26% and 0.14%, of EOs, respectively, while specimens A and B of *M*. *tomentosa* had EO contents of 0.35%, and 0.41%, respectively. These findings corroborate with those of several previous studies, which have suggested that the yields of EOs from different Myrtaceae species vary according to the studied species and the season of collection [20,21,22,23,24].

### 2.2. Chemical Composition of Essential Oils

The EOs of the specimens under study were obtained through hydrodistillation, which yielded a total of 107 chemical constituents. The hydrocarbon sesquiterpenes accounted for 70.64%, 76.79%, 66.14%, 56.74%, 75.82%, 51.2%, while the oxygenated sesquiterpenes accounted for 24.63%, 16.50%, 22.26%, 15.09%, 16.83%, 40.7% in the specimens *E. patrisii* (A, B), *E. punicifolia* (A, B), and *M. tomentosa* (A, B), respectively.

Germacrene D (20.03%), bicyclogermacrene (11.82%), and (*E*)-caryophyllene (11.04%), were identified as the major compounds in the EOs extracted from specimen A of *E. patrisii*. This is in contrast to the findings reported by Silva et al. [25] wherein (2*E*, 6*E*)-farnesol (34.5%), (2*E*, 6*Z*)-farnesol (23.2%), and a mixture of caryophylla-4(12),8(13)-dien-5α-ol, and caryophylla-4(12),8(13)-dien-5β-ol (15.6%) were identified as the major compounds in the EOs extracted from a specimen of *E. patrisii* collected in São Geraldo do Araguaia, Pará-Brazil. Germacrene D has been described in the literature as having antimicrobial properties [26]. Besides, both germacrene D and (*E*)-caryophyllene have immunomodulatory activity in human neutrophils, inhibiting Ca^2+^ mobilization, chemotaxis, and production of reactive oxygen species (ROS) [27]. This sesquiterpene constituent also has antioxidant and cytotoxic activity against melanoma cancer cells, which are responsible for skin cancer, breast adenocarcinoma, and colon carcinoma [28]. Bicyclogermacrene has been associated with larvicidal activity [29] and antiviral activity against SARS-CoV-2 [30]. (*E*)-caryophyllene, on the other hand, has been reported to exhibit antiprotozoal activity against the parasite *Leishmania amazonensis*, which causes leishmaniasis [31]. Additionally, sesquiterpenes have anticonvulsant [32], antifungal [33], and anti-inflammatory properties [34].

The EOs of *E. patrisii* specimen B were characterized by γ-elemene (25.89%), germacrene B (8.11%), and (*E*)-caryophyllene (10.76%), which was slightly lower than that of specimen A. Because it has larvicidal activity against *Spodoptera litura*, γ-elemene has the potential to be developed into biopesticides for pest control [35]. The compound is highly effective against the larvae of the mosquito species *Anopheles subpictus*, *Aedes albopictus*, and *Culex tritaeniorhynchus*, as well as having antioxidant activity and cytotoxic activity against melanoma cells [36,37]. The antibiotic [37] and antiproliferative activity [38] of Germacrene B has also been reported in the literature.

The hydrocarbon sesquiterpenes β-elemene (25.12%), (*E*)-caryophyllene (13.11%), selin-11-en-4α-ol (9.16%), and bicyclogermacrene (9.88%) were the major constituents *E. punicifolia* EOs extracted from specimen A. The main constituents of specimen B EOs were (*E*) -caryophyllene (11.47%), β-pinene (5.86%), bicyclogermacrene (5.86%), and γ-muurolene (5.55%). A sample of *E. punicifolia*, collected in the municipality of Maracanã, Pará, revealed a predominance of (*E*)-caryophyllene (9.87%), bicyclogermacrene (8.75%), and (*E*)-β-ocimene (5.50%). Low concentrations of β-pinene and γ-muurolene were also discovered at 3.91% and 2.08%, respectively [39]. Sesquiterpenes were also found to be the predominant constituents in a sample of *E*. *punicifolia* collected from the Atlantic Forest in Rio de Janeiro by Ramos et al. [40]. Conversely, the EOs extracted from *E*. *punicifolia* specimens in this study were found to be primarily composed of α-cadinol (10.6%), 10-*epi*-γ-eudesmol (10.2%), and paradisiol (9%). The use of sesquiterpenes can help patients with squamous cell carcinoma of the esophagus have a better prognosis. Furthermore, it has the potential to reduce the adverse effects of chemoradiotherapy [41]. δ-cadinene was also found in the EOs of *E. patrisii* and *E. punicifolia*, specimens A and B, at low concentrations (1.39–6.64%), and has been reported for acaricidal activity against *Psoroptes cuniculi* [42] as well as antimicrobial activity [43].

The EOs of *M. tomentosa*, specimen A, was characterized by the presence of the hydrocarbon sesquiterpenes γ-elemene (12.52%), germacrene D (11.45%), and (*E*)-caryophyllene (10.22%). For specimen B, the oxygenated sesquiterpene spathulenol had the highest concentration (40.70%), followed by the hydrocarbon sesquiterpenes α-zingiberene (9.58%) and γ-elemene (6.89%), which is significantly different from the findings previously reported in the literature [44]. Spathulenol has been reported to harbor insecticidal, insect repellent, antioxidant, anti-inflammatory, antiproliferative, and antimycobacterial properties [45,46,47,48], while α-zingiberene is a known inhibitor for aflatoxin and fungal mycotoxins as well as having antihyperlipidemic and anti-inflammatory properties [49,50]. 

#### Multivariate Analysis

**Figure 1:**
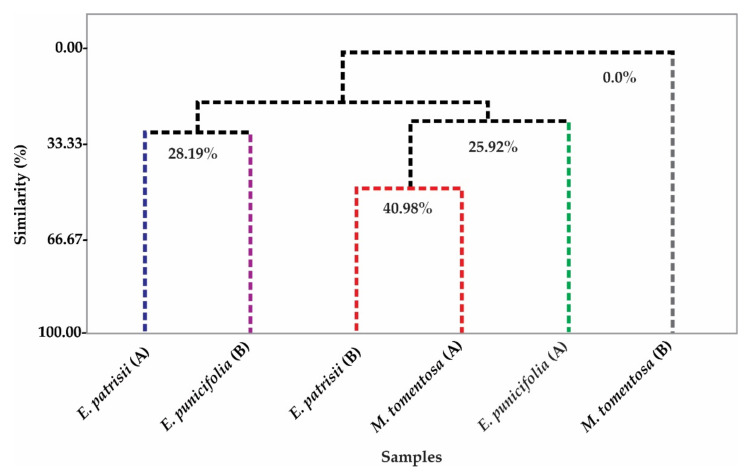
Dendrogram representing the similarity ratio of samples of EO from the leaves of *E. patrisii* (A), *E. patrisii* (B), *E. punicifolia* (A), *E. punicifolia* (B), *M. tomentosa* (A), and *M. tomentosa* (B).

To assess the similarity between the EO samples obtained by hydrodistillation, hierarchical cluster analysis (HCA) (Figure 1) was applied to the chemical compounds identified and quantified by CG/MS and CG-FID. The HCA shows that the samples *E*. *patrisii* (B) and *M*. *tomentosa* (A) have the greatest similarity (40.98%), sufficient to form a group. The chemical composition of the EOs was found to be directly influenced by the collection periods of the Amazon winter and summer samples, with a strong influence on compounds with concentrations ≥3% (Table 1).

### 2.3. Antioxidant Activity

The EOs from specimen A of *E*. *patrisii* showed inhibition of 31.4% (ABTS•+) and 99.0% (DPPH•) (Table 2). Conversely, EOs from specimen B showed inhibition of 17.9% (ABTS•+) and 204.0% (DPPH•) (Table 2). While specimens A and B had a lower antioxidant capacity than Trolox (ABTS•+), in the DPPH assay, specimens A and B had shown an antioxidant capacity equivalent to that of the Trolox standard, with specimen B exhibiting better antioxidant activity than Trolox. The profound antioxidant activity observed in specimen B may be associated with the high content of sesquiterpenes present in its chemical composition. 

The EOs extracted from specimen A of *E. punicifolia* showed inhibition of 9.5% (ABTS•+) and 408.0% (DPPH•), while that of specimen B showed inhibition of 37.7% (ABTS•+) and 285.0% (DPPH•) (Table 2). In the ABTS•+ assay, specimen B showed a higher oxidizing capacity compared to specimen A but was lower than that of the Trolox standard. In the DPPH• assay, specimen A exhibited a superior antioxidant capacity compared to both Trolox standard and specimen B. This may be attributed to the abundance of cyclic sesquiterpene compounds such as Germacrene D and (*E*)-caryophyllene in specimen A of *E. punicifolia.*, reported to offer strong antioxidant and free radicals neutralizing properties in earlier studies [54].

The EO of specimen A of *M*. *tomentosa* inhibited ABTS • and DPPH• + by 53.6% and 213%, respectively (Table 2). The inhibition of ABTS•+ and DPPH• in specimen B of the aforementioned species was 0.333% and 208.5%, respectively (Table 2). In both assays, specimen A of *M*. *tomentosa* outperformed specimen B in terms of antioxidant activity. This is the first study to report the antioxidant activity of EOs extracted from *E*. *punicifolia* and *M*. *tomentosa.* Furthermore, specimen A of *E*. *punicifolia* exhibited higher antioxidant activity in the DPPH assays compared to the other specimens studied. This may be attributed to the presence of oxygenated compounds in their composition, as DPPH is more sensitive to polar substances [55]. Additionally, synergistic action between the chemical constituents may have contributed to the higher antioxidant activity observed [56].

## 3. Materials and Methods

### 3.1. Botanical Material

The aerial parts of *E. patrisii*, *E. punicifolia*, and *M*. *tomentosa* were collected in May (A) and September (B) 2019, during the Amazon winter and summer, respectively. These collections took place in the Vila Nova district, located in the municipality of Magalhães Barata, in the State of Pará, Brazil (0°48′7.1″ S 47°33′50.3″ W). All samples were collected from fertile plants, and incorporated into the Herbário MG João Murça Pires collection of aromatic plants at the Museu Paraense Emílio Goeldi, Belém, Pará, Brazil. They were subsequently recorded as *E*. *patrisii* A (MG237487) and B (MG237497), *E. punicifolia* A (MG237519), B (MG237496), and *M*. *tomentosa* A (MG237518) and B (MG237478).

### 3.2. Preparation of Botanical Material

The leaves of *E. patrisii*, *E. punicifolia,* and *M. tomentosa* were dried for 5 days at 35 °C in an oven with air circulation before being crushed in a knife mill. The moisture content was analyzed using an ID50 infrared humidity determiner in the temperature range of 60–180 °C, with a 1 °C increment and bidirectional RS-232 °C output.

### 3.3. Essential Oil Isolation

The samples were hydrodistilled for 3 h in a modified Clevenger-type glass system coupled to a refrigeration system, to maintain condensation water at ~12 °C [57]. After extraction, the oils were centrifuged at 3000 rpm for 5 min, dehydrated with anhydrous sodium sulfate (Na_2_SO_4_), and centrifuged again under the same conditions. The oil yield was calculated as mL/100 g. The collected EOs were stored in amber glass ampoules, sealed with flame, and stored in a freezer at −15 °C. The EO yield was calculated on a dry basis (db) [58].

### 3.4. Chemical Composition Analysis

The chemical compositions of the EOs of *E*. *patrisii*, *E*. *punicifolia,* and *M*. *tomentosa*, were analyzed using a Shimadzu QP-2010 (Kyoto, Japan) plus gas chromatography system equipped with an Rtx-5MS capillary column (Restek Corporation, Bellefonte, PA USA) (30 m × 0.25 mm; 0.25 μm film thickness) coupled to a mass spectrometer (GC/MS) (Shimadzu, Kyoto, Japan). The program temperature was maintained at 60–240 °C at a rate of 3 °C/min, with an injector temperature of 250 °C, helium as the carrier gas (linear velocity of 32 cm/s, measured at 100 °C) and a splitless injection (1 μL of a 2:1000 hexane solution) using the same operating conditions as described in the literature [59,60]. Except for the carrier hydrogen gas, the components were quantified using gas chromatography (CG) on a Shimadzu QP-2010 system (Kyoto, Japan), equipped with a flame ionization detector (FID), under the same operating conditions as before. The retention index for all volatile constituents was calculated using a homologous series of n-alkanes (C_8_–C_40_) Sigma-Aldrich (San Luis, AZ, USA), according Van den Dool and Kratz [61]. The components were identified by comparison i) of the experimental mass spectra with those compiled in libraries (reference) and ii) their retention indices to those found in the literature [51,52,53].

### 3.5. Antioxidant Capacity Equivalent to Trolox

The antioxidant potential of the substances under investigation was calculated by comparing them to Trolox (6-hydroxy-2,5,7,8-tetramethylchromono-2-carboxylic acid; Sigma-Aldrich; 23881-3; São Paulo / Brazil), a water-soluble synthetic analog of vitamin E. The trolox equivalent antioxidant capacity (TEAC) was determined according to the methodology adapted from [62] modified by [63]. TEAC was based on the antioxidant inhibition of the radical cation ABTS+•. ABTS+• is a blue-green chromophore formed by the reaction between 2,2′-azino-bis (3-ethylbenzothiazoline-6-sulfonic acid) diammonium salt (ABTS; Sigma-Aldrich; A1888; São Paulo / Brazil) and potassium persulfate (K_2_O_8_S_2_; Sigma-Aldrich; 216224; São Paulo/Brazil). When antioxidants are added to this preformed cation radical, it is reduced to ABTS on a scale that depends on the antioxidant capacity, antioxidant concentration, and reaction time. Both TEAC and DPPH assays were used to determine the antioxidant capacities of the specimens’ EOs. Trolox (1 mM) was used as a standard for the calibration curves (TEAC: concentration = [absorbance + 0.0023] / 0.4162, r = 0.9789; DPPH: Concentration = [Absorbance + 0.0046] / 0.1346, r = 0.9851), and the antioxidant capacity was expressed as a percentage of inhibition.

The cuvette was first filled with 2970 μL of the ABTS+ working solution, followed by the first reading (T0). Subsequently, 30 μL of the sample was transferred to a cuvette containing the ABTS+• radical, and the second reading (T5) was recorded after 5 min. The reaction was measured using spectrophotometry by observing the change in absorbance at 734 nm for 5 min (Spectrophotometer; Bioespectro SP22; São Paulo /Brazil). Thus, the total antioxidant activity of the sample was determined, and its relationship to the reactivity of Trolox as a standard was calculated through the realization of a standard curve under the same conditions.

### 3.6. Antioxidant Capacity by the DPPH Method

The antioxidant capacity of the EOs was assessed according to the method proposed by Blois [64]. This method evaluates the ability of synthetic or natural substances to eliminate or neutralize 1,1-diphenyl-2-picrylhydrazyl (DPPH •; Sigma-Aldrich; D9132; São Paulo /Brazil) free and stable free radical. The free radical, purple or violet in color, exhibits absorbance in 515–520 nm, in ethanol or methanol solution. An antioxidant can donate a hydrogen atom or transfer an electron to the DPPH radical•, resulting in its reduced form DPPH-H, which is a stable diamagnetic molecule. This is accompanied by the loss of violet color over time to pale yellow or light violet. The change in color from dark violet to light violet, resulting from a decrease in the absorbance of the DPPH radical•, was monitored using a UV/visible spectrophotometer (517 nm; Spectrophotometer; Biospectrum SP22; São Paulo /Brazil) to determine the antioxidant capacity of the EOs. A standard curve was constructed using Trolox as a standard curve.

### 3.7. Statistical Analysis

The results are expressed as the average of three repetitive assessments ± the standard deviation. The activity of EOs from *E. patrisii*, *E. punicifolia*, and *M. tomentosa* leaves was analyzed by the Student′s *t*-test, with a *p*-value < 0.05.

#### Multivariate Analysis

Multivariate analysis was performed according to the methodology described by Silva et al. [65] and de Oliveira et al. [66], using the Minitab 17^®^ software (free version number 17, Minitab Inc., State College, PA, USA). The chemical constituents of the EOs extracted from the leaves of *E. patrisii* (A), *E. patrisii* (B), *E. punicifolia* (A), *E. punicifolia* (B), *M. tomentosa* (A), and *M. tomentosa* (B) (≥0.3%), were affixed as the experimental variables, thus forming a matrix of 6 (samples) × 26 (variables). The Euclidean distance options were used for distance measurement in the HCA of the samples, and the connection method was complete.

## 4. Conclusions

The chemical composition of the studied species was not found to differ significantly, which can be explained by the location and collection periods. The chemical profile of the EOs of the studied specimens, characterized by the terpenic class, showed a predominance of hydrocarbon sesquiterpenes β-elemene, (*E*)-caryophyllene, bicyclogermacrene, germacrene D, and γ-elemene), and oxygenated sesquiterpenes spathulenol and selin-11-4α-ol. The chemical composition of the EOs studied was not significantly influenced by the climate at the time of sample collection, as evident from the cluster analysis of the experimental variables. The results of the antioxidant activity suggested that the Myrtaceae specimens, assessed in the study, may be natural sources of antioxidants. The differences in the chemical profiles of the EOs influenced the antioxidant potential of the specimens. Specimens A of *E. punicifolia* and *E. patrisii* showed the highest and lowest antioxidant capacities, respectively, using the DPPH method. In the TEAC method, specimens A and B of *M. tomentosa* showed the highest and lowest antioxidant potentials, respectively. The antioxidant activity of the main compounds found in the EOs of the specimens has not been reported in the literature. However, the observed antioxidant effect may be due to a synergistic action between the various components.

## Figures and Tables

**Table 1:** Chemical composition of EOs extracted from leaves of *Eugenia patrisii*, *E. punicifolia*, and *Myrcia tomentosa* by (HD) hydrodistillation; concentration values are expressed in (%).

			*E. patrisii*		*E. punicifolia*		*M. tomentosa*	
RI_L_	RI_C_	Constituents	A	B	A	B	A	B
932 ^a^	932	α-Pinene	-	-	0.11	4.35	-	-
974 ^a^	974	β-Pinene	-	-	0.11	5.86	-	-
988 ^a^	989	Myrcene	-	-	0.08	-	-	-
1001 ^a^	1016	δ-2-Carene	-	-	0.01	0.03	-	-
1025 ^a^	1028	Sylvestrene	-	-	0.15	0.66	-	-
1026 ^a^	1030	1,8-Cineole	-	-	0.12	-	-	-
1032 ^a^	1036	(*Z*)*-*β-Ocimene	-	-	1.11	1.98	-	-
1044 ^a^	1048	(*E*)-β-Ocimene	-	-	3.09	4.96	-	-
1054 ^a^	1057	γ-Terpinene	-	-	0.02	0.06	-	-
1086 ^a^	1088	Terpinolene	-	-	-	0.09	-	-
1095 ^a^	1099	Linalool	-	-	0.03	0.06	-	-
1128 ^a^	1128	*allo*-Ocimene	-	-	0.36	1	-	-
1174 ^a^	1177	Terpinen-4-ol	-	-	0.05	0.1	-	-
1186 ^a^	1190	α-Terpineol	-	-	0.04	0.52	-	-
1335 ^a^	1339	δ-Elemene	3.31	0.66	1.42	3.5	4.57	-
1345 ^a^	1351	α-Cubebene	0.08	0.09	0.03	0.16	0.03	-
1373 ^a^	1374	α-Ylangene	-	0.08	-	0.12	0.06	-
1374 ^a^	1375	Isoledene	-	-	0.01	0.05	-	-
1374 ^a^	1379	α-Copaene	3.38	1.61	0.44	1.77	0.35	-
1387 ^a^	1387	β-Bourbonene	0.59	0.53	-	0.24	0.31	-
1389 ^a^	1394	β-Elemene	3.01	5.52	25.12	2.38	5.79	1.73
1409 ^a^	1413	α-Gurjunene	-	-	-	0.26	-	-
1411 ^a^	1417	*cis*-α-bergamotene	0.14	-	-	-	-	-
1417 ^a^	1424	(*E*)-Caryophyllene	11.04	10.76	13.11	11.47	10.22	4.27
1430 ^a^	1432	β–Copaene	1.05	-	0.29	0.97	0.86	-
1434 ^a^	1435	γ-Elemene	0.29	25.89	-	4.22	12.52	6.89
1439 ^a^	1442	Aromadendrene	0.46	-	0.52	-	0.42	-
1437 ^a^	1446	α-Guaiene	-	0.65	-	1.06	-	-
1447 ^c^	1447	Isogermacrene D	0.31	-	-	-	-	-
1442 ^a^	1455	6,9-Guaiadiene	-	0.2	-	0.37	0.31	-
1451 ^a^	1453	*trans*-Muurola-3,5-diene	0.2	-	-	0.61	-	-
1440 ^a^	1457	(*Z*)-β-Farnesene	-	-	-	-	-	0.96
1452 ^a^	1457	α-Humulene	3.29	2.48	3.88	4.41	2.21	-
1458 ^a^	1464	*allo*-Aromadendrene	-	-	-	0.34	0.47	-
1464 ^a^	1464	9-*epi*-(*E*)- Caryophyllene	0.38	-	-	-	-	-
1460 ^a^	1466	dehydro-Aromadendrane	-	-	0.14	-	-	-
1471 ^a^	1473	4,5-di-*epi*-Aristolochene	-	0.04	0.09	-	-	-
1471 ^a^	1477	Dauca-5,8-diene	0.2	-	-	0.13	-	-
1475 ^a^	1477	*trans*-Cadina-1(6),4-diene	-	-	-	0.74	-	-
1475 ^a^	1479	γ-Gurjunene	-	0.79	2	-	-	-
1478 ^a^	1480	γ- Muurolene	-	1.4	-	5.55	-	-
1479 ^a^	1484	*ar*-Curcumene	-	-	-	-	-	18.54
1476 ^a^	1487	β-Chamigrene	-	0.27	-	-	-	-
1484 ^a^	1487	Germacrene D	20.03	-	2.05	-	11.45	0.13
1489 ^a^	1490	β–Selinene	0.56	1.77	4.96	1.02	1.34	-
1492 ^a^	1494	δ-Selinene	0.46	0.63	-	0.96	-	-
1492 ^a^	1494	*cis*-β-Guaiene	-	-	-	-	0.99	-
1493 ^a^	1496	α- Zingiberene	-	-	-	-	-	9.58
1498 ^a^	1499	α-Selinene	-	2.87	-	-	-	-
1500 ^a^	1501	Bicyclogermacrene	11.82		9.88	5.86	6.4	-
1509 ^a^	1502	α-Bulnesene	-	0.44	-		-	-
1500 ^a^	1503	α- Muurolene	-	-	-	1.04	1.33	-
1511 ^a^	1509	δ-Amorphene	-	1.19	-		1.33	-
1502 ^a^	1510	*trans*-β-Guaiene	-	-	-	1.62	-	-
1505 ^a^	1510	β–Bisabolene	2.33	-	-	-	-	1.83
1505 ^a^	1514	(*E,E*)-α-Farnesene	0.07	-	-	-	-	-
1513 ^a^	1517	γ-Cadinene	-	0.28	-	0.63	0.41	-
1520 ^a^	1521	7-*epi*-α-selinene	-	1.56	0.31	0.29	-	-
1521 ^a^	1525	β–Sesquiphellandrene	-	-	-	-	-	3.22
1522 ^a^	1527	δ-Cadinene	6.64	1.39	1.76	4.01	3.54	-
1528 ^a^	1529	Zonarene	-	-	-	0.33	-	-
1529 ^a^	1534	(*E*)- γ-Bisabolene	0.84	0.53	-	-	0.45	3.15
1533 ^a^	1537	*trans*-Cadina-1,4-diene	-	-	0.08	0.52	0.36	-
1537 ^a^	1540	α-Cadinene	0.16	0.4	0.05	-	-	-
1540 ^b^	1542	Selina-4(15),7(11)-diene	-	3.58	-	0.72	1.21	-
1546 ^b^	1546	Selina-3,7(11)-diene	-	2.16	-	0.29	0.58	-
1556 ^a^	1549	(*E*)-Dauca-4(11),7-diene	-	0.12	-	-	-	-
1548 ^a^	1553	Elemol	-	0.51	-	-	-	-
1554 ^a^	1558	β-Vetivenene	-	0.33	-	-	-	-
1559 ^a^	1564	Germacrene B	-	8.11	-	1.1	8.31	0.9
1561 ^a^	1565	*(E*)-Nerolidol	0.37	-	-	-	-	-
1570 ^a^	1575	Dendrolasin	-	0.35	-	0.06	-	-
1577 ^a^	1582	Spathulenol	5.37	-	5.43	2.24	2.04	40.7
1582 ^a^	1587	Caryophyllene oxide	-	1.62	-	-	-	-
1590 ^a^	1589	Globulol	3.69	-	-	3.53	2.87	-
1592 ^a^	1591	Viridiflorol	1.68	0.31	3.86	2.38	2.6	-
1596 ^a^	1594	Fokienol	-	0.56	-	-	-	-
1608 ^a^	1600	β-Atlantol	-	0.15	-	-	-	-
1595 ^a^	1602	Cubeban-11-ol	-	-	0.7	-	-	-
1600 ^a^	1606	Rosifoliol	0.38	-	0.43	0.61	0.39	-
1608 ^a^	1613	Humulene epoxide II	0.3	-	0.22	-	-	-
1630 ^a^	1623	γ-Eudesmol	-	2.52	-	-	-	-
1618 ^a^	1626	Junenol	-	-	0.06	-	0.48	-
1629 ^a^	1629	Eremoligenol	0.38	-	-	-	-	-
1627 ^a^	1634	1-*epi*-Cubenol	-	-	0.21	-	-	-
1635 ^a^	1636	*cis*-Cadin-4-en-7-ol	0.17	0.52		0.38	-	-
1645 ^a^	1638	Cubenol	-	-	0.11	-	2.74	-
1639 ^a^	1640	*allo*-Aromadendrene epoxide	-	0.06	-	-	-	-
1640 ^a^	1647	*epi-*α-muurolol	4.09	-	-	-	-	-
1642 ^a^	1648	Selina-3,11-dien-6*α*-ol	-	0.73	-	-	-	-
1652 ^a^	1650	Himachalol	1.13	0.32	0.14	-	-	-
1644 ^a^	1655	α-Muurolol	-	-	-	3.15	-	-
1651 ^a^	1659	Pogostol	-	1.95	-	-	-	-
1652 ^a^	1660	α-Cadinol	7.12	-	1.91	2.44	4.38	-
1668 ^a^	1666	14-hydroxy-9-*epi-(E*)-Caryophyllene	-	0.07	-	-	-	-
1658 ^a^	1669	Selin-11-en-4α-ol	-	-	9.16	-	-	-
1658 ^a^	1670	*neo*-Intermedeol	-	0.48	-	-	-	-
1670 ^a^	1671	Bulnesol	-	-	-	0.34	-	-
1687 ^a^	1681	Eudesma-4(15)-dien-1β-ol	-	0.16	-	-	-	-
1685 ^a^	1686	α-Bisabolol	0.05	-	-	-	-	-
1679 ^a^	1692	Khusinol	-	-	0.03	-	-	-
1700 ^a^	1700	Eudesm-7(11)-en-4-ol	-	2.34	-	-	1.29	-
1709 ^a^	1706	Mayurone	-	0.08	-	-	-	-
1708 ^a^	1713	*cis*-Thujopsenal	-	0.12	-	-	-	-
1714 ^a^	1717	Nootkatol	-	1.93	-	-	-	-
1775 ^a^	1773	2-α-hydroxy-Amorpha-4,7(11)-diene	-	2.18	-	-	-	-
2026 ^a^	2030	(*E,E*)-Geranyl linalool	-	-	-	-	0.04	-
		Hydrocarbon monoterpenes	-	-	5.04	18.99	-	-
		Oxygenated monoterpenes	-	-	0.24	0.68	-	-
		Hydrocarbon sesquiterpenes	70.64	76.79	66.14	56.74	75.82	51.2
		Oxygenated sesquiterpenes	24.63	16.50	22.26	15.09	16.83	40.7
		Oxygenated diterpenes	-	-	-	-	0.04	-
		Others	-	-	-	0.04	-	-
		Total	95.37	93.29	93.68	91.54	92.65	91.9

RI_C_: calculated from a series of *n*-alkanes (C_8_–C_40_) in a DB-5MS column capillar column, RI_L_): Literature ^a^ Adams [51], ^b^ Mondello [52] and ^c^ Nist [53].

**Table 2:** Activity of elimination of the radicals ABTS•+ and DPPH• (%) of EOs from leaves of the specimens of *Eugenia* and *Myrcia*.

Species	Specimen	Collection Period	CA-ABTS•+ (%)	CA-DPPH• (%)
*Eugenia patrisii*	A	May	31.4 ± 0.1	99.0 ± 0.099
	B	September	17.9 ± 0.069	204.0 ± 0.877
*Eugenia punicifolia*	A	May	9.5 ± 0.034	408.0 ± 0.10
	B	September	37.7 ± 0.035	285.0 ± 0.028
*Myrcia tomentosa*	A	May	53.6 ± 0.150	213.0 ± 0.905
	B	September	0.333 ± 0.247	208.5 ± 0.940

Values are expressed as mean and standard deviation (*n* = 3) of the percentage of inhibition.

## Sample Availability

Samples of *Eugenia patrisii* Vahl, *E. punicifolia* (Kunth) DC., and *Myrcia tomentosa* (Aubl.) DC. The essential oil of the Museu Paraense Emílio Goeldi is available from the authors.

## References

[1] Cutrim E.S.M., Teles A.M., Mouchrek A.N., Filho V.E.M., Everton G.O. (2019). Evaluation of Antimicrobial and Antioxidant Activity of Essential Oils and Hydroalcoholic Extracts of Zingiber officinale (Ginger) and Rosmarinus officinalis (Rosemary). Revista Virtual de Química.

[2] Pavela R. (2015). Essential oils for the development of eco-friendly mosquito larvicides: A review. Ind. Crop. Prod..

[3] Mohamed A.A., Behiry S.I., Ali H.M., El-Hefny M., Salem M.Z., Ashmawy N.A. (2020). Phytochemical Compounds of Branches from P. halepensis Oily Liquid Extract and S. terebinthifolius Essential Oil and Their Potential Antifungal Activity. Process.

[4] Gyesi J.N., Opoku R., Borquaye L.S. (2019). Chemical Composition, Total Phenolic Content, and Antioxidant Activities of the Essential Oils of the Leaves and Fruit Pulp of *Annona muricata* L. (Soursop) from Ghana. Biochem. Res. Int..

[5] De Oliveira M.S., Silva S.G., da Cruz J.N., Ortiz E., da Costa W.A., Bezerra F.W.F., Cunha V.M.B., Cordeiro R.M., de Jesus Chavez Neto A.M., de Aguiar Andrade E.H., Inamuddin R.M., Asiri A.M. (2019). Supercritical CO2 Application in Essential Oil Extraction. Industrial Applications of Green Solvents—Volume II.

[6] Bakkali F., Averbeck S., Idaomar M. (2008). Biological effects of essential oils—A review. Food Chem. Toxicol..

[7] Ali M.M., Yusuf M.A., Abdalaziz M.N. (2017). GC-MS Analysis and Antimicrobial Screening of Essential Oil from Lemongrass (Cymbopogon citratus). Int. J. Pharm. Chem..

[8] Govaerts R., Sobral M., Ashton P., Barrie F., Holst B.K., Landrum L.L., Matsumoto K., Mazine F.F., Niclughadha E., Proença C. World checklist of Myrtaceae. Royal Botanic Gardens, Kew. http://apps.kew.org/wcsp/2014.

[9] Proença C.E.B., Amorim B.S., Antonicelli M.C., Bünger M., Burton G.P., Caldas D.K.D., Costa I.R., Faria J.E.Q., Fernandes T., Gaem P.H. Myrtaceae in Flora do Brasil -Jardim Botânico do Rio de Janeiro. http://floradobrasil.jbrj.gov.br/reflora/floradobrasil/FB171.

[10] Cascaes M.M., Guilhon G.M.S.P., Andrade E.H.D.A., Zoghbi M.D.G.B., Santos L.D.S. (2015). Constituents and Pharmacological Activities of Myrcia (Myrtaceae): A Review of an Aromatic and Medicinal Group of Plants. Int. J. Mol. Sci..

[11] Da Silva V.P., Alves C.C.F., Miranda M.L.D., Bretanha L.C., Balleste M.P., Micke G.A., Silveira E.V., Martins C.H.G., Ambrosio M.A.L.V., Silva T.D.S. (2018). Chemical composition and in vitro leishmanicidal, antibacterial and cytotoxic activities of essential oils of the Myrtaceae family occurring in the Cerrado biome. Ind. Crop. Prod..

[12] Singh H.P., Kaur S., Negi K., Kumari S., Saini V., Batish D.R., Kohli R.K. (2012). Assessment of in vitro antioxidant activity of essential oil of Eucalyptus citriodora (lemon-scented Eucalypt; Myrtaceae) and its major constituents. LWT.

[13] Figueiredo P.L.B., Fernandes H.A., Da Silva A.R.C., Alves N.S.F., Setzer W.N., Da Silva J.K.R., Maia J.G.S. (2019). Variability in the Chemical Composition of Eugenia biflora Essential Oils from the Brazilian Amazon. Nat. Prod. Commun..

[14] Chaieb K., Hajlaoui H., Zmantar T., Ben Kahla-Nakbi A., Rouabhia M., Mahdouani K., Bakhrouf A. (2007). The chemical composition and biological activity of clove essential oil, Eugenia caryophyllata (*Syzigium aromaticum* L. Myrtaceae): A short review. Phytother. Res..

[15] Mazine F.F., Valdemarin K.S., Bünger M., Faria J.E.Q., Fernandes T., Giaretta A., Santana K.C., Sobral M., Souza M.A.D. Eugenia in Flora do Brasil. Jardim Botânico do Rio de Janeiro. http://floradobrasil.jbrj.gov.br/reflora/floradobrasil/FB10338.

[16] De Sousa E.M., dos Anjos T.O., do Nascimento L.D., de Andrade E.H.A., Costa C.M.L., de Faria L.J.G. (2019). Cinética de secagem e composição química da polpa do fruto de eugenia patrisii vahl. (myrtaceae). Impactos das Tecnologias na Engenharia Química 2.

[17] Sales D.S., Carmona F., De Azevedo B.C., Taleb-Contini S.H., Bartolomeu A.C.D., Honorato F.B., Martinez E.Z., Pereira A.M.S. (2014). Eugenia punicifolia(Kunth) DC. as an Adjuvant Treatment for Type-2 Diabetes Mellitus: A non-Controlled, Pilot Study. Phytother. Res..

[18] Basting R.T., Nishijima C.M., Lopes J.A., Santos R.C., Périco L.L., Laufer S., Bauer S., Costa M.F., Santos L.C., Rocha L.R. (2014). Antinociceptive, anti-inflammatory and gastroprotective effects of a hydroalcoholic extract from the leaves of Eugenia punicifolia (Kunth) DC. in rodents. J. Ethnopharmacol..

[19] Santos M.F., Amorim B.S., Burton G.P., Fernandes T., Gaem P.H., Lourenço A.R.L., Lima D.F., Rosa P.O., Santos L.L.D., Staggemeier V.G. Myrcia in Flora do Brasil. Jardim Botânico do Rio de Janeiro. http://floradobrasil.jbrj.gov.br/reflora/floradobrasil/FB10660.

[20] Montalván M., Peñafiel M.A., Ramírez J., Cumbicus N., Bec N., Larroque C., Bicchi C., Gilardoni G. (2019). Bec Chemical Composition, Enantiomeric Distribution, and Sensory Evaluation of the Essential Oils Distilled from the Ecuadorian Species Myrcianthes myrsinoides (Kunth) Grifo and Myrcia mollis (Kunth) DC (Myrtaceae). Plants.

[21] Raposo J.D.A., Figueiredo P.L.B., Santana R.L., Junior A.Q.D.S., Suemitsu C., da Silva R., Mourão R.H.V., Maia J.G.S. (2018). Seasonal and circadian study of the essential oil of Myrcia sylvatica (G. Mey) DC., a valuable aromatic species occurring in the Lower Amazon River region. Biochem. Syst. Ecol..

[22] Da Costa J.S., Barroso A.S., Mourão R.H.V., Da Silva J.K.R., Maia J.G.S., Figueiredo P.L.B. (2020). Seasonal and Antioxidant Evaluation of Essential Oil from Eugenia uniflora L., Curzerene-Rich, Thermally Produced in Situ. Biomolecules.

[23] Tietbohl L.A.C., Lima B.G., Fernandes C.P., Santos M.G., Silva F.E.B., Denardin E.L.G., Bachinski R., Alves G.G., Sil-va-Filho M.V., Rocha L. (2012). Comparative study and anticholinesterasic evaluation of essential oils from leaves, stems and flowers of Myrciaria floribunda (H.West ex Willd.) O. Berg. Lat. Am. J. Pharm..

[24] Scalvenzi L., Grandini A., Spagnoletti A., Tacchini M., Neill D., Ballesteros J.L., Sacchetti G., Guerrini A. (2017). Myrcia splendens (Sw.) DC. (syn. M. fallax (Rich.) DC.) (Myrtaceae) Essential Oil from Amazonian Ecuador: A Chemical Characterization and Bioactivity Profile. Molecules.

[25] Da Silva J.K.R., Andrade E.H.A., Barreto L.H., Da Silva N.C.F., Ribeiro A.F., Montenegro R.C., Maia J.G.S. (2017). Chemical Composition of Four Essential Oils of Eugenia from the Brazilian Amazon and Their Cytotoxic and Antioxidant Activity. Medicines.

[26] Zheljazkov V.D., Kacaniova M., Dincheva I., Radoukova T., Semerdjieva I.B., Astatkie T., Schlegel V. (2018). Essential oil composition, antioxidant and antimicrobial activity of the galbuli of six juniper species. Ind. Crop. Prod..

[27] Schepetkin I.A., Özek G., Özek T., Kirpotina L.N., Khlebnikov A.I., Quinn M.T. (2020). Chemical Composition and Immunomodulatory Activity of *Hypericum perforatum* Essential Oils. Biomolecules.

[28] Casiglia S., Bruno M., Bramucci M., Quassinti L., Lupidi G., Fiorini D., Maggi F. (2017). Kundmannia sicula (L.) DC: A rich source of germacrene D. J. Essent. Oil Res..

[29] Huong L.T., Huong T.T., Huong N.T., Hung N.H., Dat P.T., Luong N.X., Ogunwande I.A. (2020). Mosquito Larvicidal Activity of the Essential Oil of Zingiber collinsii against Aedes albopictus and Culex quinquefasciatus. J. Oleo Sci..

[30] Narkhede R.R., Pise A.V., Cheke R.S., Shinde S.D. (2020). Recognition of Natural Products as Potential Inhibitors of COVID-19 Main Protease (Mpro): In-Silico Evidences. Nat. Prod. Bioprospecting.

[31] Moreira R.R.D., Dos Santos A.G., Carvalho F.A., Perego C.H., Crevelin E.J., Crotti A.E.M., Cogo J., Cardoso M.L.C., Nakamura C.V. (2019). Antileishmanial activity of Melampodium divaricatum and Casearia sylvestris essential oils on Leishmania amazonensis. Revista do Instituto de Medicina Tropical de São Paulo.

[32] De Oliveira C.C., de Oliveira C.V., Grigoletto J., Ribeiro L.R., Funck V.R., Grauncke A.C.B., de Souza T.L., Souto N.S., Furian A.F., Menezes I.R.A. (2016). Anticonvulsant activity of β-caryophyllene against pentylenetetrazol-induced seizures. Epilepsy Behav..

[33] Karpiński T.M. (2020). Essential Oils of Lamiaceae Family Plants as Antifungals. Biomolecules.

[34] Brito L.F., Oliveira H.B.M., Selis N.D.N., E Souza C.L.S., Júnior M.N.S., De Souza E.P., Da Silva L.S.C., Nascimento F.D.S., Amorim A.T., Campos G.B. (2019). Anti-inflammatory activity of β -caryophyllene combined with docosahexaenoic acid in a model of sepsis induced by Staphylococcus aureus in mice. J. Sci. Food Agric..

[35] Benelli G., Govindarajan M., AlSalhi M.S., Devanesan S., Maggi F. (2017). High toxicity of camphene and γ-elemene from Wedelia prostrata essential oil against larvae of *Spodoptera litura* (Lepidoptera: Noctuidae). Environ. Sci. Pollut. Res..

[36] Govindarajan M., Rajeswary M., Senthilmurugan S., Vijayan P., Alharbi N.S., Kadaikunnan S., Khaled J.M., Benelli G. (2017). Curzerene, trans-β-elemenone, and γ-elemene as effective larvicides against Anopheles subpictus, Aedes albopictus, and Culex tritaeniorhynchus: Toxicity on non-target aquatic predators. Environ. Sci. Pollut. Res..

[37] Da Silva J.K.R., Pinto L., Burbano R., Montenegro R.C., Guimaraes E.F., Andrade E.H.A., Maia J.G.S. (2014). Essential oils of Amazon Piper species and their cytotoxic, antifungal, antioxidant and anti-cholinesterase activities. Ind. Crop. Prod..

[38] Alves C.C.F., Oliveira J.D., Estevam E.B.B., Xavier M.N., Nicolella H.D., Furtado R.A., Tavares D.C., Miranda M.L.D. (2020). Antiproliferative activity of essential oils from three plants of the Brazilian Cerrado: *Campomanesia adamantium* (Myrtaceae), *Protium ovatum* (Burseraceae) and *Cardiopetalum calophyllum* (Annonaceae). Braz. J. Biol..

[39] Pereira R.A., Zoghbi M.D.G.B., Bastos M.D.N.D.C. (2010). Essential Oils of Twelve Species of Myrtaceae Growing Wild in the Sandbank of the Resex Maracanã, State of Pará, Brazil. J. Essent. Oil Bear. Plants.

[40] Ramos M.F.D.S., Monteiro S.D.S., Da Silva V.P., Nakamura M.J., Siani A.C. (2010). Essential Oils From Myrtaceae Species of the Brazilian Southeastern Maritime Forest (Restinga). J. Essent. Oil Res..

[41] Chang Z., Gao M., Zhang W., Song L., Jia Y., Qin Y. (2017). Beta-elemene treatment is associated with improved outcomes of patients with esophageal squamous cell carcinoma. Surg. Oncol..

[42] Guo X., Shang X., Li B., Zhou X.Z., Wen H., Zhang J. (2017). Acaricidal activities of the essential oil from Rhododendron nivale Hook. f. and its main compund, δ-cadinene against Psoroptes cuniculi. Veter Parasitol..

[43] Pérez-López A., Cirio A.T., Rivas-Galindo V.M., Aranda R.S., De Torres N.W. (2011). Activity againstStreptococcus pneumoniaeof the Essential Oil and δ-Cadinene Isolated fromSchinus molleFruit. J. Essent. Oil Res..

[44] Sá F.A.S., Borges L.L., Paula J.A.M., Sampaio B.L., Ferri P.H., Paula J.R. (2012). Essential oils in aerial parts of Myrcia tomentosa: Composition and variability. Rev. Bras. Farm..

[45] Benelli G., Pavela R., Drenaggi E., Desneux N., Maggi F. (2020). Phytol, (E)-nerolidol and spathulenol from Stevia rebaudiana leaf essential oil as effective and eco-friendly botanical insecticides against *Metopolophium dirhodum*. Ind. Crop. Prod..

[46] De Oliveira M.A.S., Coutinho H.D.M., Neto L.J.D.L., De Oliveira L.C.C., Da Cunha F.A.B. (2020). Repellent activity of essential oils against culicids: A review. Sustain. Chem. Pharm..

[47] Dzul-Beh A.D.J., García-Sosa K., Uc-Cachón A.H., Bórquez J., Loyola L.A., Barrios-García H.B., Peña-Rodríguez L.M., Molina-Salinas G.M. (2019). In vitro growth inhibition and bactericidal activity of spathulenol against drug-resistant clinical isolates of Mycobacterium tuberculosis. Rev. Bras. Farm..

[48] Nascimento K.F.D., Moreira F.M.F., Santos J.A., Kassuya C.A.L., Croda J.H.R., Cardoso C.A.L., Vieira M.D.C., Ruiz A.L.T.G., Foglio M.A., de Carvalho J.E. (2018). Antioxidant, anti-inflammatory, antiproliferative and antimycobacterial activities of the essential oil of Psidium guineense Sw. and spathulenol. J. Ethnopharmacol..

[49] Nerilo S.B., Rocha G.H.O., Tomoike C., Mossini S.A.G., Grespan R., Mikcha J.M.G., Machinski M. (2015). Antifungal properties and inhibitory effects upon aflatoxin production byZingiber officinaleessential oil inAspergillus flavus. Int. J. Food Sci. Technol..

[50] Li J., Thangaiyan R., Govindasamy K., Wei J. (2020). Anti-inflammatory and anti-apoptotic effect of zingiberene on isoproterenol-induced myocardial infarction in experimental animals. Hum. Exp. Toxicol..

[51] Adams R.P., Adams R.P. (2007). Identification of Essential Oil Components by Gas Chromatography/Mass Spectroscopy.

[52] Mondello L. FFNSC 2 Flavors and Fragrances of Natural and Synthetic Compounds 2 (Mass Spectral Database) 2011. https://www.wiley.com/en-us/Mass+Spectra+of+Flavors+and+Fragrances+of+Natural+and+Synthetic+Compounds%2C+3rd+Edition-p-9781119069843.

[53] Stein S., Mirokhin D., Tchekhovskoi D., Mallard G., Mikaia A., Zaikin V., Sparkmanm D. The NIST mass spectral search program for the nist/epa/nih mass spectra library. Standard Reference Data Program of the National Institute of Standards and Technology, Gaithersburg, MD, US, 2011. https://www.nist.gov/system/files/documents/srd/Ver20Man.pdf.

[54] Victoria F.N., Lenardão E.J., Savegnago L., Perin G., Jacob R.G., Alves D., da Silva W.P., Motta A.D.S.D., Nascente P.D.S. (2012). Essential oil of the leaves of Eugenia uniflora L.: Antioxidant and antimicrobial properties. Food Chem. Toxicol..

[55] Gatto L.J., Fabri N.T., De Souza A.M., Da Fonseca N.S.T., Furusho A.D.S., Miguel O.G., Dias J.D.F.G., Zanin S.M.W., Miguel M.D. (2020). Chemical composition, phytotoxic potential, biological activities and antioxidant properties of Myrcia hatschbachii D. Legrand essential oil. Braz. J. Pharm. Sci..

[56] Nafis A., Kasrati A., Jamali C.A., Mezrioui N., Setzer W., Abbad A., Hassani L. (2019). Antioxidant activity and evidence for synergism of Cannabis sativa (L.) essential oil with antimicrobial standards. Ind. Crop. Prod..

[57] Gurgel E.S.C., de Oliveira M.S., Souza M.C., da Silva S.G., de Mendonça M.S., Filho A.P.D.S.S. (2019). Chemical compositions and herbicidal (phytotoxic) activity of essential oils of three Copaifera species (Leguminosae-Caesalpinoideae) from Amazon-Brazil. Ind. Crop. Prod..

[58] Ferreira O.O., Da Cruz J.N., Franco C.D.J.P., Silva S.G., Da Costa W.A., De Oliveira M.S., Andrade E.H.D.A. (2020). First Report on Yield and Chemical Composition of Essential Oil Extracted from Myrcia eximia DC (Myrtaceae) from the Brazilian Amazon. Molecules.

[59] De Oliveira M.S., Da Cruz J.N., Da Costa W.A., Silva S.G., Brito M.D.P., De Menezes S.A.F., Neto A.M.D.J.C., Andrade E.H.D.A., Junior R.N.D.C. (2020). Chemical Composition, Antimicrobial Properties of *Siparuna guianensis* Essential Oil and a Molecular Docking and Dynamics Molecular Study of its Major Chemical Constituent. Molecules.

[60] Silva S.G., de Oliveira M.S., Cruz J.N., da Costa W.A., da Silva S.H.M., Maia A.A.B., de Sousa R.L., Junior R.N.C., Andrade E.H.D.A. (2021). Supercritical CO2 extraction to obtain Lippia thymoides Mart. & Schauer (Verbenaceae) essential oil rich in thymol and evaluation of its antimicrobial activity. J. Supercrit. Fluids.

[61] Van Den Dool H., Kratz P.D. (1963). A generalization of the retention index system including linear temperature programmed gas—liquid partition chromatography. J. Chromatogr. A.

[62] Miller M., Rao J., Wlodawer A., Gribskov M.R. (1993). A left-handed crossover involved in amidohydrolase catalysis. FEBS Lett..

[63] Re R., Pellegrini N., Proteggente A., Pannala A., Yang M., Rice-Evans C. (1999). Antioxidant activity applying an improved ABTS radical cation decolorization assay. Free Radic. Biol. Med..

[64] Blois M.S. (1958). Antioxidant Determinations by the Use of a Stable Free Radical. Nat. Cell Biol..

[65] Silva S.G., Figueiredo P.L.B., Nascimento L.D., Da Costa W.A., Maia J.G.S., Andrade E.H.A. (2018). Planting and seasonal and circadian evaluation of a thymol-type oil from Lippia thymoides Mart. & Schauer. Chem. Central J..

[66] De Oliveira M.S., da Silva V.M.P., Freitas L.C., Silva S.G., Cruz J.N., Andrade E.H.D.A. (2021). Extraction Yield, Chemical Composition, Preliminary Toxicity of Bignonia nocturna (Bignoniaceae) Essential Oil and in Silico Evaluation of the Interaction. Chem. Biodivers..

